# Should joint lavage be performed in the treatment of juvenile canine spontaneous/haematogenous septic arthritis?

**DOI:** 10.18849/ve.v10i1.699

**Published:** 2025-02-20

**Authors:** Philippa Wells, Paul Aldridge

**Keywords:** arthritis, arthropathy, canine, dogs, haematogenous, lavage, septic, spontaneous

## Abstract

**PICO question:**

In dogs less than 18 months old with spontaneous/haematogenous bacterial septic arthritis, how effective is treatment with joint lavage and antimicrobial therapy, compared to antimicrobial therapy alone?

**Clinical bottom line:**

**Category of research:**

Treatment.

**Number and type of study designs reviewed:**

One case series of 5 dogs.

**Strength of evidence:**

Weak.

**Outcomes reported:**

Successful clinical outcomes were reported in patients both with and without joint lavage.

**Conclusion:**

No conclusions can be made regarding the impact of joint lavage on haematogenous septic arthritis in juvenile dogs as excellent outcomes were achieved in patients with and without lavage. However appropriate antibiosis continues to be pertinent with one case persistently infected when inappropriate antibiotics were selected.

## How to apply this evidence in practice

The application of evidence into practice should take into account multiple factors, not limited to: individual clinical expertise, patient’s circumstances and owners’ values, country, location or clinic where you work, the individual case in front of you, the availability of therapies and resources.

Knowledge Summaries are a resource to help reinforce or inform decision-making. They do not override the responsibility or judgement of the practitioner to do what is best for the animal in their care.

### Clinical Scenario

A juvenile canine patient is confirmed to have haematogenous/spontaneous septic arthritis, should you perform joint lavage before starting antibiotics or will an antibiotic course alone be sufficient?

## The evidence

Following a literature search on two databases, only one study met the inclusion criteria for this Knowledge Summary question. Several papers were excluded, as per the criteria, as they did not discriminate cases with regard to septic arthritis aetiologies, where one may anticipate different treatment requirements and responses. Additionally, the one available study (Fitch et al., 2003) includes a small sample size of 5 cases, further limiting the strength of the evidence. Two of the five patients had joint lavage performed, whilst the other three were prescribed antibiotics alone, with additional variation in the antimicrobial selection and duration.

On this basis no conclusions can be drawn on whether joint lavage improves outcomes of haematogenous septic arthritis cases in juvenile dogs where there is no predisposing or underlying cause.

## Summary of the evidence

**Fitch Et al. (2003):** Hematogenous Septic Arthritis in the Dog: Results of Five Patients Treated Nonsurgically With Antibiotics Aim: Retrospective study evaluating the effectiveness of nonsurgical treatment using antibiotics to treat hematogenous septic arthritis in five dogs.

Population	Dogs with a diagnosis of haematogenous septic arthritis from Colorado State University (USA) and Louisiana State University (USA) between 1994 and 2000. Patients were between 2 and 9 months old, with 3 giant breed dogs, 1 large breed dog, and 1 medium breed dog. Patients were excluded if they had any previous or pre-existing surgery or trauma to the affected limb, or if they did not have a clinical outcome reported for at least 12 months.
Sample Size	5 dogs.
Intervention Details	All 5 dogs had arthrocentesis performed with decompression of the joint. The synovial fluid was sent for culture in 3/5 cases, with a positive culture result in all 3 samples. Two-needle joint lavage was performed in two cases (2 and 3), with 1–3 l of saline. Empirical antibiotics were started immediately in all patients. Following culture results, the antibiotic choice was changed in all 3 cultured cases (1, 3, and 4). Case 1: Amoxicillin-clavulanate, 15 mg/kg q12 hours, for 10 days Cultured *staphylococcus intermedius* Cephalexin, 22 mg/kg q8 hours, for 21 days. Case 2: Two-needle joint lavage Cephalexin, 22 mg/kg q8 hours, for 42 days No culture performed. Case 3: Two-needle joint lavage Cefazolin, 20 mg/kg q3 hours, for 1 day Cultured *Streptococcus B-haemolytic spp* Cephalexin, 21 mg/kg q6 hours, for 30 days. Case 4: Amoxicillin-clavulanate, 13 mg/kg q12 hours, for 56 days Cultured *Pasteurella multocida* Enrofloxacin, 7.5 mg/kg q12 hours, for 20 days. Case 5: Amoxicillin-clavulanate, 14 mg/kg q12 hours, for 42 days No culture performed.
Study design	Retrospective case series
Outcome studied	Subjective assessments included: Reported timing of improvement and resolution of clinical signs. Clinical signs included: lameness, synovial effusion, arthralgia, and distal limb oedema. A owner interview and questionnaire were completed, at least one year post infection, in all cases.
Main findings (relevant to PICO question)	Long term outcome was assessed by owner questionnaire, at which persistent lameness was reported in cases 1 and 4. The bacterium cultured in case 4 was not sensitive to either of the chosen antibiotics. Case 1 was treated with an initial 10-day antibiotic course, following which there was recurrence of clinical signs and a second antibiotic course was started. An excellent outcome, according to resolution of clinical signs, was reported in cases 2, 3, and 5. Four owners (Cases 1, 2, 3, and 5) reported an improvement of clinical signs within 3 days of starting the antibiotic course, and complete resolution of clinical signs within one month.
Limitations	There is a small sample size, which may limit the relevance to a broader population. The treatment courses were heterogeneous, with different empirical treatment choices and differing course durations. Due to the retrospective nature of the paper these decisions could not be fully understood. Only two patients were treated with joint lavage and antibiotics, and 3 patients with antibiotics alone which is insufficient to make a comparison between the two groups. There is additionally no justification for whether they elected to lavage the affected joint or not. Two owners declined culture of the synovial fluid limiting assessment of whether the empirical antibiotics were appropriate, however both cases had an ‘excellent’ outcome reported. There were only subjective outcomes recorded, with no objective measurements. There is no validated questionnaire utilised to measure outcomes. Whilst long term follow up is useful, in this paper it relied on owners recalling the time frame for improvement and clinical resolution over a year later which may lead to inaccuracies.

Table note...

## Appraisal, application and reflection

A literature search of two databases found no papers with a direct comparison of treatment protocols of antibiotic treatment and joint lavage, or antibiotic treatment alone. This Knowledge Summary appraised one small case series (Fitch et al., 2003) describing the treatment and outcomes of five cases. Whilst case series sit low in the hierarchy of evidence, the limited size in this study further diminishes the evidentiary value of this paper. Additionally, this case series included a heterogenous population, where culture was not routinely performed, which further limits the comparison of outcomes.

Whilst larger studies on septic arthritis exist (Marchevsky & Read, (1999), Mielke et al., (2018), Clements et al., (2005), Phillips & Bleyaert, (2021)), they do not differentiate juvenile patients from older animals with pre-existing joint pathology and include patients with varying aetiologies (including penetrating injuries) and patients with peri-articular surgical implants. Due to the ability of an implant to act as a nidus for infection, explantation is required in these cases to allow resolution of clinical signs: therefore, these alternative aetiologies have been excluded from this Knowledge Summary.

There were six patients from Clements et al. (2005) who were 12 months old or younger. However, four of these patients had previous peri-articular surgery of the affected joint which may impact the course of their disease and treatment. The two remaining cases, which both involved the elbow joint and underwent needle lavage, recovered with no report of recurrence; however, there was no control group treated with antibiotics alone within the same demographic to allow comparison and it is difficult to draw conclusions from two cases.

Due to the lack of specific data within this age group and aetiology, consideration may be given to studies of septic arthritis treatment that cover a broader age range and a mixture of aetiologies. Studies by Clements et al. (2005), Mielke et al., (2018), and Phillips & Bleyaert (2021) all report no difference in outcome between cases treated with antibiotics alone and those that also underwent joint lavage and/or arthrotomy.

## Methodology

At this time the current literature provides weak evidence to indicate whether juvenile canine patients with haematogenous septic arthritis treated with joint lavage and antimicrobial therapy have an improved outcome compared to those treated with antimicrobials alone.

**Search Strategy:** 

Databases searched and dates covered	CAB Abstracts on the OVID interface; 1973 to October 2023 PubMed accessed via the NCBI website; date of coverage 1920 to October 2023
Search terms	Cab Abstracts: (dog or dogs or bitch or bitches or canine or canines) ((Spontaneous or haematological or haematogenous or hematological or hematogenous or bacterial or septic or infect*) and (arthritis or arthropathy)) (flush or flushing or lavage or nonsurgical* or non-surgical* or ‘non surgical*’ or medical or conservative) (antibiotic* or antimicrobial* or antibacterial*or anti-microbial*) 1 and 2 and 3 and 4 Pubmed: (dog or dogs or bitch or bitches or canine or canines) ((Spontaneous or haematological or haematogenous or hematological or hematogenous or bacterial or septic or infect*) and (arthritis or arthropathy)) (flush or flushing or lavage or nonsurgical* or non-surgical* or medical or conservative) (antibiotic or antimicrobial or antibacterial or anti-microbial) 1 and 2 and 3 and 4
Dates searches performed:	27 October 2023

**Exclusion / Inclusion criteria:** 

Exclusion	Case report. Opinion pieces. Papers not relating to septic arthritis. Papers not relating to dogs. Papers that do not include outcomes.
Inclusion	Papers that include signalment, treatment and clinical outcome of all cases reported.

**Search outcome:** 

Database	Number of results	Excluded – case report	Excluded – opinion piece	Excluded – non- spontaneous cause	Excluded – not dogs	Excluded – not septic arthritis	Excluded – not answering PICO	Total relevant papers
CAB Abstracts	15	0	1	11	2	0	0	1
PubMed	63	4	0	10	19	28	1	1
Total relevant papers when duplicates removed								1

## ORCID

Philippa Wells: https://orcid.org/0009-0001-2509-1637

Paul Aldridge: https://orcid.org/0000-0002-3805-2610

## Conflict of interest

The authors declare no conflicts of interest.

## Disclaimer

Knowledge Summaries are a peer-reviewed article type which aims to answer a clinical question based on the best available current evidence. It does not override the responsibility of the practitioner. Informed decisions should be made by considering such factors as individual clinical expertise and judgement along with patient’s circumstances and owners’ values. Knowledge Summaries are a resource to help inform and any opinions expressed within the Knowledge Summaries are the author’s own and do not necessarily reflect the view of the RCVS Knowledge. Authors are responsible for the accuracy of the content. While the Editor and Publisher believe that all content herein are in accord with current recommendations and practice at the time of publication, they accept no legal responsibility for any errors or omissions, and make no warranty, express or implied, with respect to material contained within. For further information please refer to our Terms of Use.
